# Supplementary material for: A preclinical model of post-surgery secondary bone healing for subtrochanteric femoral fracture based on fuzzy interpretations
Source: PLoS One. 2022 Jul 21;17(7):e0271061. doi: 10.1371/journal.pone.0271061 (PMC9302822; doi:10.1371/journal.pone.0271061)
Supplement: S1 File — (DOCX) [file pone.0271061.s001.docx]

**ANSYS APDL script (Batch mode) for FE analysis**

RESUME,'DHS_1','dbb','C:\Users\pratik\Desktop\Jr_4_analysis'

/PREP7

*dele,MatF

*dele,POF

*dele,Elem

*dele,EARRAY

*dim,MatF,array,262288,1

*VREAD,MatF,'MatF','csv','C:\Users\pratik\Desktop\Jr_4_analysis'

(F12.0)

*dim,POF,array,262288,1

*VREAD,POF,'POF','csv','C:\Users\pratik\Desktop\Jr_4_analysis'

(F12.0)

*dim,Elem,array,262288,1

*VREAD,Elem,'Elem','csv','C:\Users\pratik\Desktop\Jr_4_analysis'

(F12.0)

*do,i,1,262288,1

MP,EX,7,MatF(i,1)

MP,NUXY,7,POF(i,1)

esel,s,elem,,Elem(i,1)

eplot

EMODIF,all,mat,7

*enddo

allsel

eplot

FDELE,ALL,ALL

DDELE,ALL,ALL

/INPUT,'Loadst1_updated','mac','C:\Users\pratik\Desktop\Jr_4_analysis',, 0

allsel

/solu

SOLVE

/prep7

esel,s,type,,7

eplot

/post1

etable,strain1,EPEL,1

etable,strain2,EPEL,2

etable,strain3,EPEL,3

etable,volume,VOLU

*GET, Ecount,elem,0,count

*DIM,earray,ARRAY,Ecount,4

e1=0

*do,i,1,Ecount,1

e1=elnext(e1)

earray(i,1)=e1

*GET,earray(i,1), ETAB, 1,elem, e1

*GET,earray(i,2), ETAB, 2,elem, e1

*GET,earray(i,3), ETAB, 3,elem, e1

*GET,earray(i,4), ETAB, 4,elem, e1

*enddo

*CFOPEN,'stimuli_calc','txt','C:\Users\pratik\Desktop\Jr_4_analysis'

*VWRITE,earray(1,1),earray(1,2),earray(1,3),earray(1,4)

(F16.8,' ',F16.8,' ',F16.8,' 'F16.8)

*cfclose

SAVE,'DHS_1','db','C:\Users\pratik\Desktop\Jr_4_analysis'

FINISH

/EXIT,ALL

**MATLAB script for fuzzy logic**

for k=1:50

E = load('D:\Pratik_Jr4_trial\stimuli_calc.txt');

E0= (1/3)*(E(:,1)+E(:,2)+E(:,3));

Y0=(1/sqrt(2)*(sqrt((((E(:,1))-(E(:,2))).^2)+(((E(:,1))-(E(:,3))).^2)+(((E(:,2))-(E(:,3))).^2))));

t = 262288;

fis=Fuzzy15;

DC=[];

for i=1:t

C = evalfis(fis,[E0(i) Y0(i) P(i) Car(i) B(i) Pa(i) Ba(i)]);

DC=vertcat(DC,C);

end

delCP=DC(:,1);

delCC=DC(:,2)./100;

delCB=DC(:,3)./100;

delCcar=DC(:,2);

delCBone=DC(:,3);

CC=CC+delCC;

CB=CB+delCB;

P=P+delCP;

Car=Car+delCcar;

B=B+delCBone;

delCPa=zeros(t,1);

delCBa=zeros(t,1);

for i=1:t

for j=2:6

delCPa(X(j,i),1)=delCPa(X(j,i),1)+(delCP(X(1,i),1))*(0.48/Y(j,i));

end

end

for i=1:t

for j=2:6

delCBa(X(j,i),1)=delCBa(X(j,i),1)+(delCBone(X(1,i),1))*(0.48/Y(j,i));

end

end

Pa=Pa+delCPa;

Ba=Ba+delCBa;

Pa(Pa>100)=100;

Ba(Ba>100)=100;

P(P>100)=100;

Car(Car>100)=100;

B(B>100)=100;

Pa(Pa<0)=0;

Ba(Ba<0)=0;

P(P<0)=0;

Car(Car<0)=0;

B(B<0)=0;

CC(CC>1)=1;

CB(CB>1)=1;

CC(CC<0)=0;

CB(CB<0)=0;

CT=1-(CB+CC);

MatF0=zeros(t,1);

POF0=zeros(t,1);

for i=1:t

MatF0(i)=((4000*CB(i))+(200*CC(i))+((3)*CT(i)));

POF0(i)=(((0.3)*(CB(i)))+((0.45)*(CC(i)))+((0.3)*(CT(i))));

end

MatF=mean(MatF0);

POF=mean(POF0);

itr_val =num2str(k);

an_cal = 'EMF';

name=strcat(an_cal,itr_val,'.xlsx');

xlswrite(name,MatF0,'A1');

writematrix(MatF,'D:\Pratik_Jr4_trial\MatF.csv');

writematrix(POF,'D:\Pratik_Jr4_trial\POF.csv');

save(['healing6' num2str(k) '.mat']')

end

**Calling ANSYS APDL script in MATLAB for integrating FE analysis with fuzzy logic**

system('"C:\Program Files\ANSYS Inc\v150\ansys\bin\winx64\ANSYS150.exe" -b -m 24000 -i D:\Pratik_Jr4_trial\APDL_script17.mac -o D:\Pratik_Jr4_trial\APDL_script_out.mac')
