# Supplementary material for: A preclinical model of post-surgery secondary bone healing for subtrochanteric femoral fracture based on fuzzy interpretations
Source: PLoS One. 2022 Jul 21;17(7):e0271061. doi: 10.1371/journal.pone.0271061 (PMC9302822; doi:10.1371/journal.pone.0271061)
Supplement: Attachment — Submitted filename: Response to Reviewers.docx [file pone.0271061.s002.docx]

**Point-to-point Response to the Reviewer’s Comments**

**Manuscript Number:** PONE-D-21-37870

**Old Title:** Secondary bone healing for subtrochanteric femoral fracture treated with locking and dynamic compression plates: a comparative phenomenological assessment based on fuzzy interpretations

**New Title:** Phenomenological prediction of post-surgery secondary bone healing for subtrochanteric femoral fracture based on fuzzy interpretations

**Authors:** Pratik Nag, Souptick Chanda

The authors thankfully acknowledge the recommendation of the Academic Editor to revise the manuscript by addressing the corrections suggested by the reviewers. The authors are also indebted to the esteemed reviewers for their positive feedback and constructive criticisms of the work, which have altogether significantly helped in improving the quality of the manuscript.

The authors, hereby, submit the revised manuscript for re-review as a full-length article. The revised manuscript, along with the point-to-point response to the reviewer’s comments, is submitted accordingly.

All changes in the revised manuscript are highlighted in “YELLOW”.

**Response to the Reviewers:**

**Reviewer 1**

**General Comments**

In the manuscript by Nag and coworkers, the authors have developed a theoretical assessment of secondary bone healing when treated with either locking plates or dynamic compression plates. The authors use fuzzy logic-based interpretations to comparatively asses the two methods of treatment. But the methods and results are not well described. Considering the broad readership of Plos One, the authors should describe the methods and their results clearly. Overall, the language used in the manuscript is very ambiguous and makes the manuscript difficult to read. The authors have predicted the healing of the bone by simulating the mechanical compression of the bone using finite element modeling and tissue differentiation using fuzzy logic methods. But these predictions are not supported by any experiments. Without any experimental data, the claims of the authors are not well supported. I do not recommend this manuscript for publication in the current form. But if the authors make significant changes in the text and add some experimental data, the manuscript could add to the current understanding of bone healing.

**Response:** The authors would like to thank the reviewer for the valuable feedback which helped a great deal in enhancing the quality of the manuscript. Also, the authors gratefully acknowledge the suggestions/concerns with regard to incorporation of experimental as well as statistical data to validate the predictive model. However, it is important to clarify here that an experimental validation of the present bone healing model is tantamount to carrying out an animal study at the least, if not a clinical trial, which is going to be a project in itself. Nevertheless, the authors unequivocally agree to the proposition that a statistical comparison is necessary vis-a-vis the existing literature on bone healing progression. Also, a mechanical validation of the *in silico* bone model is warranted such that the mechanical input parameters responsible for bone healing may be compared in toto. It may also be noted that the plated constructs were virtually curated and hence, physical models of the implants are not available. In line with the suggestion, however, a study involving the intact bone (model# 3406, Sawbones Inc.) is incorporated in the manuscript which correlates the force-displacement characteristics between the experiment and finite element (FE) models subjected to identical compressive loading (Fig. 1). The regression analysis and student’s t-test, revealed a significant correlation between measured and FE predicted curves. A high value of correlation coefficient (R = 0.99) and low standard error of estimate (SE = 0.02 mm) along with a regression slope, b~1.0 (PE of b = 1.4%), and intercept of a = 0.022 were predicted for the intact femur (95% confidence interval). This imparted confidence in the efficacy of the FE model generation scheme which was further employed to develop the plated constructs (LP and DCP).

Fig.1: Experimental validation of the intact femur under static compression loading

The following paragraph is now added under Materials and Method section (Page no. 6-7, line: 28-7) along with Fig. 1 (Fig. 2d in the revised manuscript):

***“The FE models of intact femur was validated based on force-displacement characteristics obtained under in vitro loading (Fig. 2d). For experimental test, the intact bone specimen was potted inside a custom-made aluminium rig, after removing the condylar section, as shown in Fig. 2d. The custom-made fixture was introduced in order to provide sufficient distal constraint. Identical loading and boundary condition were ensured for validation of the FE model. The regression analysis and student’s t-test, revealed a significant correlation between measured and FE predicted curves. A high value of correlation coefficient (R = 0.99) and low standard error of estimate (SE = 0.02 mm) along with a regression slope, b~1.0 (PE of b = 1.4%), and intercept of a = 0.022 were predicted for the intact femur (95% confidence interval). This imparted confidence in the efficacy of the FE model generation scheme which was further employed to develop the plated constructs (LP and DCP).”***

In order to further substantiate our bone healing results, the interfragmentary movements (IFM) at different healing intervals were compared with that reported by two different studies – one numerical mechanobiology based study Lacroix et al., 2002 and the other an animal experiment carried out by Claes et al. (Fig. 2). Whereas the numerical model demonstrated identical trend in bone healing progression as predicted by our scheme, a significant drop in IFM was measured in the animal experiment as the bone healing progressed beyond 4^th^ week post-operatively. It may, however, be noted that the starting point of the IFM characteristics varied significantly owing perhaps to the differences in model and mechanical input parameters (stimulation). This further illustrates the dependence of the bone healing progression on the input parameters, e.g., mechanical strain. Hence, carrying out an animal study will require precise estimation/measurement of the loading regime such that a full-fledged validation can be contemplated. This poses another challenge for simulation and subsequent validation of the scheme. Nonetheless, a future study is being envisaged to address this aspect.

Fig. 2: The predicted IFM values in LP compared with existing literature.

The following sentences are now added in the discussion section of revised manuscript (page no.13, line 8-17). Also, Fig. 2 (Fig. 10b in the revised manuscript) is added now in order to substantiate our predicted data:

***“The interfragmentary movements (IFM) at different healing intervals were compared with that reported by two different studies – one numerical mechanobiology based study Lacroix et al., 2002 [63] and the other an animal experiment carried out by Claes et al. [64] (Fig. 10b). Whereas the numerical model [63] demonstrated identical trend in bone healing progression as predicted by our scheme, a significant drop in IFM was measured in the animal experiment [64] as the bone healing progressed beyond 4th week post-operatively. It may, however, be noted that the starting point of the IFM characteristics varied significantly owing perhaps to the differences in model and mechanical input parameters (stimulation). This further illustrates the dependence of the bone healing progression on the input parameters, e.g., mechanical strain.”***

**References:**

***[63]*** ***Lacroix D, Prendergast PJ. A mechano-regulation model for tissue differentiation during fracture healing: analysis of gap size and loading. J Biomech. 2002 Sep;35(9):1163-71. doi: 10.1016/s0021-9290(02)00086-6.***

***[64]*** ***Claes L, Augat P, Suger G, Wilke HJ. Influence of size and stability of the osteotomy gap on the success of fracture healing. J Orthop Res. 1997 Jul;15(4):577-84. doi: 10.1002/jor.1100150414.***

Understanding the criticality of the above insight as reflected by the esteemed reviewer, the authors further decided to add the following sentence in the manuscript (Page no. 14, line:7-9) to highlight the pertinent limitation of the predictive scheme:

***“Therefore, animal study is warranted at the least, with precise estimation of the loading regime such that a full-fledged validation of the present scheme can be contemplated.”***

The authors would also like to state here that the manuscript has been checked thoroughly for any ambiguity in language. If found, the same has been revised for better readability.

**Major comments:**

1. The title of the manuscript is very long and confusing. The authors should make their title concise and clear. They can use the short title as their main title.

**Response:** The authors agree to the concern raised by the esteemed reviewer and do feel that the title in its current form may arouse confusion. Accordingly, the title of the manuscript is changed.

***Old title:*** *Secondary bone healing for subtrochanteric femoral fracture treated with locking and dynamic compression plates: a comparative phenomenological assessment based on fuzzy interpretations*

***New title:*** *Phenomenological prediction of post-surgery secondary bone healing for subtrochanteric femoral fracture based on fuzzy interpretations*

2. In the second paragraph of introduction, the author state that “The biomechanical environment achieved using LP is totally different form that obtained through DCP”. But they do not provide any references for this statement and they also do not elaborate on this statement.

**Response:** The authors are thankful to the reviewer for this suggestion. Accordingly, the reference and following sentence is added in page no. 3, line 19-21:

***“The biomechanical environment achieved using LP is totally different from that obtained through DCP [4]. The surrounding environment of the screws in DCP is governed by a mix of effects contrary to LP, whose mechanics is simple like that of unilateral external fixation device.”***

**Reference:**

***[4] Perren SM. Evolution of the internal fixation of long bone fractures. The scientific basis of biological internal fixation: choosing a new balance between stability and biology. J Bone Joint Surg Br. 2002;84(8):1093-1110. doi:10.1302/0301-620x.84b8.13752***

3. In the introduction the authors do not highlight a clear gap in the current understanding, making it difficult to assess the importance of their work. The authors should clearly state the hypothesis at the end of the introduction

**Response:** The authors agree to the concern of the reviewer. However, it may be noted that no modelling approach addressed the influence of screw type in determining the overall progression of secondary fracture healing. This is highlighted in the last paragraph of the introduction along with the hypothesis (Page no. 4-5, Line 28-8).

However, the authors realize that the hypothesis in present form does not reflect appropriately on the importance of the fuzzy scheme proposed. Hence, the following sentence is now added in the last paragraph of Introduction section of the revised manuscript to highlight the said importance (Page no. 5, Line 5-8):

**“*****The study is based on a hypothesis that fuzzy logic controls could actually be used to predict tissue transformation in a realistic and non-invasive manner for secondary healing in plated bone.”***

4. In the biological part of the methods section, the authors write about fuzzy logic based methods. But they do not explain “Mamdami type fuzzy logic controller” and do not provide any reference. Further in the same section, they bring in the concept of Membership function. But these functions are not clearly defined and explained. Without properly explaining these functions, it is rather difficult to comprehend the results

**Response:** The authors are thankful to the reviewer for this valuable feedback. Accordingly, pertinent references are added in the revised manuscript related to “Mamdami type fuzzy logic controller” (Page no. 8; Line 8-12):

***“******Mamdani fuzzy inference was introduced to build a control system by incorporating a set of linguistic control rules obtained from experienced human operators [48]. Mamdani type fuzzy logic controller was employed to link the seven input state variables using some linguistic rules to predict (deterministically) the change in three output state variables (Fig. 3).”***

**Reference:**

***[48].*** ***Mamdani, E.H., and S. Assilian. ‘An Experiment in Linguistic Synthesis with a Fuzzy Logic Controller’. International Journal of Man-Machine Studies 7, no. 1 (January 1975): 1–13. https://doi.org/10.1016/S0020-7373(75)80002-2.***

Also, a statement along with a reference is added to explain the membership functions as follows (Page no. 9; Line no. 1-4):

***“******The membership function of a fuzzy set is apparently the indicator function in classical sets. In fuzzy logic, it describes the degree of truth as an extension of valuation [49]. Membership function characterizes the information of the fuzzy set and assigns each object a grade of membership ranging between zero and one.”***

**Reference:**

***[49].*** ***L.A. Zadeh, Fuzzy sets, Information and Control, Volume 8, Issue 3, 1965, Pages 338-353, ISSN 0019-9958, https://doi.org/10.1016/S0019-9958(65)90241-X.***

5. The text in the results section is very ambiguous. It is not clear whether the authors are describing an experimental data or a prediction of their simulation.

**Response:** The authors regret the confusion of the reviewer. The authors would like to gently clarify that the study is based on outcomes of simulation. FE analysis and fuzzy logic model were used to predict secondary bone healing process: Extending from response to the reviewer 1’s general comment, the authors would like to further clarify that good agreement is shown between animal experiment carried out previously in another work and numerical prediction of the present work. Nevertheless, in order to obviate any confusion, the sentence (Page no. 9, line: 22-25) is now rephrased as follows:

**“*Bone concentration, cartilage concentration, blood perfusion and change of modulus in the callus region were predicted over space and time for both cases*** ***of LP and DCP using FE analysis and fuzzy logic-based simulation scheme*.”**

6. The figures are not prepared carefully and the figure legends do not describe the figure well.

**Response:** The authors are thankful to the reviewer for this suggestion. Accordingly, Fig. 3 is now changed into two figures and was ensured that it fit in one page. Furthermore, in Fig. 4, Fig. 5, Fig. 6 (Fig. 5, Fig. 6, Fig. 7 in the updated manuscript) description is added below the colour bars. Also, all the plots of Fig. 8 (Fig. 9 in updated manuscript) were kept in one page in the updated manuscript.

**Minor comments:**

1. The manuscript is hard to read as the authors use passive voice throughout the manuscript. The authors should try to write more in active voice.

**Response:** The authors regret the inconvenience of the reviewer. However, we hope that the reviewer will agree that certain reporting in a technical document should best be done in passive sense. Nonetheless, the authors have revised the manuscript to the extent possible without downplaying the overall meaning/sense.

2. In figure 3b, it is not clear what is the meaning of destructive and medium.

**Response:** The authors would like to clarify that destructive, medium, or low are the linguistic terms given to various ranges of quantifying values (strain, concentration, perfusion).

3. It would be better to make the plots in figure 3 smaller, so that all the plots fit in one page. This makes it much easier to understand.

**Response:** The authors are thankful to the reviewer for this suggestion. Accordingly, Fig. 3 is now changed into two figures while ensuring that they fit in one page.

4. In Fig. 4, it is not mentioned what the color bar represents. Similarly, in Fig. 5 and Fig. 6 description of color bar is missing

**Response:** The authors are thankful to the reviewer for the suggestion. Accordingly, in Fig.4, Fig. 5, Fig. 6, (Fig. 5, Fig. 6, Fig. 7 in the updated manuscript) the description is added below the colour bars.

6. In Fig. 8, it is not mentioned whether tissue concentration and distortional strain is a mean of multiple simulations or a single simulation. It is also not clear how many simulations were performed. Moreover, it would be better if the authors bring all the plots of Fig. 8 in one page.

**Response:** The authors acknowledge the query of the reviewer. The authors would like to clarify that the tissue concentrations and distortional strain are the outcomes of iterative simulation steps taking each iteration as one day. Accordingly, the following line is added in page no. 9, line 25-27:

***“******The tissue concentrations and distortional strain are predicted based on the outcomes of iterative simulation steps taking each iteration as one day. The simulation was continued iteratively till 60 healing steps running in loop.”***

7. The authors should put more effort in organizing the figures and annotating them.

**Response:** The authors are grateful to the reviewer for helping us identifying certain flaws in organizing and annotating the figures. All figure captions are now revised and figures rearranged in a more meaningful manner. Proper annotations are made wherever necessary.

**Reviewer 2**

General comment: A well-written manuscript wherein the study predicts and compares the healing process of a subtrochanteric femoral fracture step by step in response to two different fixation techniques, the Locking Plate (LP) and the Dynamic Compression Plate (DCP), using the fuzzy logic-based iterative model. Authors here hypothesized that the different screw fixation mechanisms seen in these two techniques might influence the healing outcome differently. Final results suggested an initial delay in response with DCP but an altogether identical progression of healing for both the cases after the 8th week time-point along with an early stabilisation of the callus for LP. All these findings were in accordance with previous studies done using different tools.

Thus, this study brings to the limelight that fuzzy logic controls could actually be used to study and predict tissue transformation in a realistic and non-invasive manner. In fracture management, it can help to foresee the probable course of healing process and compare the expected outcomes with the use of different types of fixation plates prior to the surgery itself. This will help to select the best option available for the patient based on his individual characteristics and fracture types beforehand.

**Response:** The authors are thankful to the esteemed reviewer for his compliments and indebted to him for the insightful review. This will motivate us to take up further studies in this field in future.

1. Reference missing in the first paragraph of the introduction.

**Response:** The authors are grateful to the reviewer for this pertinent suggestion. As per the suggestion, the following two reference has now been added to the line in the “Introduction” section of the revised manuscript in page number 3, line no. 4 and 7:

**References:**

***[1]. Moroni A, Hoang-Kim A, Lio V, Giannini S. Current augmentation fixation techniques for the osteoporotic patient. Scand J Surg. 2006;95(2):103-9. doi: 10.1177/145749690609500205.***

***[2]. George C. Babis, Panayotis N. Soucacos, Bone scaffolds: The role of mechanical stability and instrumentation, Injury, Volume 36, Issue 4, Supplement,2005, Pages S38-S44, ISSN 0020-1383, https://doi.org/10.1016/j.injury.2005.10.009.***

2. Various authors have observed that LP performs better than DCP in older or weaker bone [1,5]. Uhl et al. found that DCP provides inherently more stability than LP [6]. Gap closure and bone-to-bone load transfer resulted in better mechanical performance of DCP over LP during axial compression [6]. The author claims various authors and cited only one. Add few more citations to claim LP better than DCP.

**Response:** The authors are thankful to the reviewer for the above observation. Accordingly, one sentence is added and four more references are cited appropriately in the revised manuscript to further substantiate the claim that LP is better than DCP (Page no. 3-4, Line: 26-2).

***“Various authors have observed that LP performs better than DCP in older or weaker bone [3,7,8]. Few other biomechanical studies found LP to have more stiffness and superior failure load compared to other implants [9,10,11]. Uhl et al. found that DCP provides inherently more stability than LP [6]. Gap closure and bone-to-bone load transfer resulted in better mechanical performance of DCP over LP during axial compression [6].”***

**References:**

***[8].*** ***Wagner M, Frenk A, Frigg R. New concepts for bone fracture treatment and the Locking Compression Plate. Surg Technol Int. 2004; 12:271-7.***

***[9].*** ***Aminian A, Gao F, Fedoriw WW, Zhang LQ, Kalainov DM, Merk BR. Vertically oriented femoral neck fractures: mechanical analysis of four fixation techniques. J Orthop Trauma. 2007 Sep;21(8):544-8. doi: 10.1097/BOT.0b013e31814b822e.***

***[10].*** ***Crist BD, Khalafi A, Hazelwood SJ, Lee MA. A biomechanical comparison of locked plate fixation with percutaneous insertion capability versus the angled blade plate in a subtrochanteric fracture gap model. J Orthop Trauma. 2009 Oct;23(9):622-7. doi: 10.1097/BOT.0b013e3181a2a31d.***

***[11].*** ***Floyd JC, O'Toole RV, Stall A, Forward DP, Nabili M, Shillingburg D, Hsieh A, Nascone JW. Biomechanical comparison of proximal locking plates and blade plates for the treatment of comminuted subtrochanteric femoral fractures. J Orthop Trauma. 2009 Oct;23(9):628-33. doi: 10.1097/BOT.0b013e3181b04835. PMID: 19897983.***

3. What was the values obtained for mesh sensitivity analysis?

**Response:** The authors would like to state that, the mesh convergence analysis was carried out with three different mesh size of 0.5 mm, 1.0 mm, and 1.5 mm constituting 427113, 303869 and 262362 elements, respectively, in the callus region. For mesh sizes 0.5 mm, 1.0 mm and 1.5 mm, the values of distortional strain were predicted to be 0.1309, 0.1311 and 0.1050. Thus, doubling the mesh size from 0.5 mm (fine sized mesh) to 1.0 mm (medium sized mesh) in the callus region resulted in change of the distortional strain by 0.1% whereas the change was almost 20% from medium sized mesh to coarse sized mesh (1.5 mm). Similarly, the variation of the peak von Mises stress between fine and medium mesh was less than 5% at identical location of callus region. Therefore, in view of computational efficiency, the medium sized mesh was considered to be sufficiently accurate. Accordingly, the following sentences are added in the revised manuscript (Page no. 6, line:3-12)

***“******A mesh convergence analysis was carried out with three different mesh size of 0.5 mm, 1.0 mm, and 1.5 mm constituting 427113, 303869 and 262362 elements respectively in the callus region. For mesh sizes 0.5 mm, 1.0 mm and 1.5 mm, the values of distortional strain were predicted to be 0.1309, 0.1311 and 0.1050. Thus, doubling the mesh size from 0.5 mm (fine sized mesh) to 1.0 mm (medium sized mesh) in the callus region resulted in change of the distortional strain by 0.1% whereas the change was almost 20% from medium sized mesh to coarse sized mesh (1.5 mm) Similarly, the variation of the peak von Mises stress between fine and medium mesh was less than 5% at identical location of callus region. Therefore, in view of computational efficiency, the medium sized mesh was considered to be sufficiently accurate.”***

4. Will there be any changes in the parameter if the loading was done cyclic rather than static loading?

**Response:** The authors deeply acknowledge the thoughtful comment of the reviewer. The nature of musculoskeletal load is cyclic/dynamic. The authors are of the opinion that clinically there can be stability issue of the bony construct if the fractured bone is subjected to cyclic loading. Computationally speaking, healing may expedite or delayed depending on the amplitude of the cyclic loading which needs to be assessed before commenting. Nonetheless, the authors recognize this as a limitation and a scope for future study and hence, was added in discussion section (Page no. 13; Line no. 23).

“***The future scope of study may include the cyclic nature of loading.***”

5. Add a paragraph on the limitation of the study and FEA.

**Response:** The authors acknowledge the suggestion of the reviewer. Though the authors did mention limitation of the study (Page no. 13, line:11-25), limitations w.r.t FE analysis were not addressed. FE analysis is an approximate method which is sensitive to parameters like loading, material properties and boundary conditions. Bone material property is complex in nature and patient specific. Mimicking the real material property is a tedious task and oftentimes infeasible. Also, the physiological load distribution in real femur is difficult to model through FE analysis. Output results may vary considerably, thereby necessitating experimental validation like the one the present study has conducted. Furthermore, the limitation of such iterative FE study is that the iterative time steps can be viewed as pseudo time steps and their correlation with real time is unknown and patient-specific. All these aspects are now addressed in one paragraph of the revised manuscript (Page no. 14, line 3-9):

***“******FE analysis is an approximate method which is sensitive to parameters like loading, material properties and boundary conditions. Output results may vary considerably, thereby necessitating experimental validation of the FE scheme, like the one the present study has conducted. Also, there exists no precise relationship between the iterative time steps involved, and actual time in days or weeks. Therefore, animal study is warranted at the least, with precise estimation of the loading regime such that a full-fledged validation of the present scheme can be contemplated.”***

6. Could you please add a note on why the tools used in this study are better than other comparison tools used elsewhere?

**Response:** The authors would like to gently state that mechanoregulatory based schemes are equation oriented and requires more computational time to predict any biological phenomenon. In contrast, rule-based models, e.g., fuzzy interpretations are relatively easier to implement and requires less computational time. Hence, such fuzzy interpretation-based tools are more computationally efficient. Furthermore, such techniques provide additional advantage of incorporating any new rule without disruption of the original model. Going forward such tools will be in high demand for predictive analysis and real time assessment of any new implant and biomechanics thereof. Accordingly, the following sentences are added (Page no. 14, line 12-15):

***“******Rule-based models, e.g. fuzzy logic tools are relatively easier to implement and require less computational time as compared to the equation-based mechanoregulatory models.*** ***Furthermore, such techniques provide additional flexibility of incorporating any new rule without disruption of the original model.”***

7. Please elaborate on the possible in-future benefits of this study?

**Response:** The authors are grateful to the reviewer for this insightful suggestion. According to the reviewer’s suggestion, the following line is added (Page no. 14, line 19-22):

“***While plan of action for traditional treatment of fractures depends primarily on the experience of the orthopaedist, the present tool can act as a platform to predict post-surgery effects in advance and, thereby help clinicians strategize the best implantation technique for a patient*.”**

8.. Could you please add a brief concluding paragraph towards the end?

**Response:** The authors most humbly thank the reviewer for the suggestion. Although a concluding paragraph stating the possibilities of the study was presented in earlier version of the manuscript, the same has now been rephrased to incorporate the possible in-future benefits and superiority of the present tool as compared to others (Page no. 14, line 10-22):

“***Nevertheless, the present preclinical scheme is robust enough to predict bone healing for different implantation techniques, and thus can serve as a non-invasive tool for evaluating the relative merits of these implants. Rule-based models, e.g. fuzzy logic tools are relatively easier to implement and require less computational time as compared to the equation-based mechanoregulatory models.*** ***Furthermore, such techniques provide additional flexibility of incorporating any new rule without disruption of the original model. After incorporating various biological and patient-specific parameters, the fuzzy logic-based iterative model can further account for different clinical phenomena, such as smoking, diabetes, etc. Additionally, the linguistic rules can easily be altered as per the understanding of the medical experts for a more rigorous preclinical assessment. While plan of action for traditional treatment of fractures depends primarily on the experience of the orthopaedist, the present tool can act as a platform to predict post-surgery effects in advance and, thereby help clinicians strategize the best implantation technique for a patient*.*”***
