# Supplementary material for: A preclinical model of post-surgery secondary bone healing for subtrochanteric femoral fracture based on fuzzy interpretations
Source: PLoS One. 2022 Jul 21;17(7):e0271061. doi: 10.1371/journal.pone.0271061 (PMC9302822; doi:10.1371/journal.pone.0271061)
Supplement: Attachment — Submitted filename: Response to Reviewers.docx [file pone.0271061.s003.docx]

**Point-to-point Response to the Reviewers’ Comments**

**Manuscript Number:** PONE-D-21-37870R1

**Old Title:** Phenomenological prediction of post-surgery secondary bone healing for subtrochanteric femoral fracture based on fuzzy interpretations

**New Title:** A preclinical model of post-surgery secondary bone healing for subtrochanteric femoral fracture based on fuzzy interpretations

**Authors:** Pratik Nag, Souptick Chanda

The authors thankfully acknowledge the recommendation of the Academic Editor to revise the manuscript by addressing the corrections suggested by the reviewers. The authors are also indebted to the esteemed reviewers for their positive feedback and constructive criticisms of the work, which have altogether significantly helped in improving the quality of the manuscript.

The authors, hereby, submit the revised manuscript for possible publication as a full-length article. The revised manuscript, along with the point-to-point response to the reviewer’s comments, is submitted accordingly.

All changes in the revised manuscript are highlighted in “YELLOW”.

**Response to the Reviewers:**

**Reviewer 1**

**General comment:** The authors have answered most of the comments. Though authors did not perform experiments, they compared their simulation data with existing experiments. As mentioned by the authors, performing experiments could be a complete work on its own. But the language used in the manuscript is still hard to comprehend. I would recommend publication only if the authors address the minor comments.

**[Response]:** The authors gratefully acknowledge the comment of the esteemed reviewer. All changes suggested have been carried out in the revised draft to the best of the authors’ knowledge and understanding.

**Minor comments:**

**Comment 1:** The figures have improved from the submitted version. But the figure legends are too short and do not describe the figures well. This particularly true for Figs. 5-9.

**[Response]:** By acknowledging the above comment, the authors too realise that some of the figure legends are not sufficiently legible. Hence, size of all figure legends (Figs. 5 – 9) are now increased considerably for better legibility. Moreover, some of the captions are revised to add more description to these figures.

**Comment 2:** Authors mostly use passive voice to describe the results. In general Passive voice is used to describe the methods. But it would be best to use active voice to describe the results. The article below uses active voice for describing results.

https://www.nature.com/articles/s41567-018-0358-7

I hope that the authors would agree that this adds to clarity of the manuscript.

**[Response]:** The authors acknowledge the above comment of the reviewer. The authors also understand and appreciate the criticality of reporting the results in a direct manner for better readability. In that sense, we agree that it is best to use active voice while reporting results in particular. Therefore, based on the suggestion of the reviewer, the Results section has been revised completely (Page 9, Lines 21-29; Page 10, Lines 10-16 & 24-26; Page 11, Lines 3-6) by changing from passive to active voice, wherever required.

We hope that the section in the present revised form is more lucid and tangible as opposed to the previous version. We also gratefully thank the reviewer for his/her thoughtful appraisal of the language, which has helped enhance the overall quality of the manuscript. All changes in the Results section have been marked in ‘Yellow’ for ready reference.

**Reviewer 2**

**General comment:** This reviewer has strong objection to the word "Phenomenological" in the title. Can this word be omitted from the title? The dictionary meaning of the word is "relating to the science of phenomena as distinct from that of the nature of being." as such it being distinct from the nature of being is misleading in the title.

**[Response]:** The authors fully subscribe to the observation of the reviewer. As per suggestion, we have now omitted the term ‘phenomenological’ and modified the title as follows:

***New Title: A preclinical model of post-surgery secondary bone healing for subtrochanteric femoral fracture based on fuzzy interpretations***

***Old Title: Phenomenological prediction of post-surgery secondary bone healing for subtrochanteric femoral fracture based on fuzzy interpretations***

**Reviewer 3**

**General comment:** To set the context of my review, I first read the original article (without previous comments or suggestions from other reviewers). I then added my own observations/impressions and finally, read the concerns raised by both reviewers, which I found sensible and needed. I finally went (after having finished my revision) onto read the amended manuscript, which reads much better than the original.

I found this manuscript interesting, and easy to follow (with a few exceptions on the language and jargon). Possibly, due to my background in both clinical and research settings with both human and animal patients, that helped me to perceive the value of this study, as long as we acknowledge that this is a ‘model’ (and modelling is very powerful and valuable in many aspect, but in some instances, clinicians they either lack the understanding of scientific literature of find hard to give credit to models since they work with ‘real’ cases).

In this work, the authors set the context of fractures (e.g. causes, importance, etc) which affects different populations. The importance of various elements for fracture healing (stability, bone healing, haemorrhage, callus formation, stages, etc) is also broke down in the context of the primary (1) and secondary (2) bone types of healing.

The key role of the position of the screws in both types of plates (DCP being the standard) is discussed, the authors introduced the new type of plate proposed and analysed in their study, i.e. locking plate (LP), and the benefits of this in regards to screw position and fixation and in comparison to DCP, arguably better than LP, according to some previous works. Also, other predictive models and their pros and cons are accurately presented and discussed in detail. To complete, they highlighted the lack of studies comparing overall progression of fracture healing derived from the effect of ‘screw type’. Outstanding is the fact that most models presented are, in ‘primitive 3D models’ (the authors’ opinion) instead of ‘real’ bone and implants.

The authors’ interest is to demonstrate the predictive power of applying non-invasive fuzzy logic controls to determine tissue transformation and (secondary) healing in plate bones. With a particular interest, as they noted, in the influence of the type of screw fixation. I here would stress that the manuscript (as I think it shows in its final form) ensures that this work is based on a ‘model’.

**[Response]:** First of all, the authors are grateful to the esteemed reviewer for his compliments. The authors also duly acknowledge the keen observation and meaningful insights from the reviewer with regards to the present study. We understand the reviewer’s position on calling the work based on a ‘model’. It is true that our assessments of the implant types were based on *in silico* models, and the bone healing predictions were based on a state-of-the-art rule-based scheme, i.e. fuzzy logic. Therefore, it is rational and justified to emphasize this aspect categorically in the ‘Title’ as well as in the ‘Abstract’. Accordingly, the title has now been modified as follows:

***New Title: A preclinical model of post-surgery secondary bone healing for subtrochanteric femoral fracture based on fuzzy interpretations***

***Old Title: Phenomenological prediction of post-surgery secondary bone healing for subtrochanteric femoral fracture based on fuzzy interpretations***

Furthermore, instead of terming our study-objective as a ‘preclinical scheme’ in the Abstract, we have now rephrased the same as ‘preclinical model’ (Page 2, Line 15). Moreover, this aspect has been maintained throughout the manuscript, wherever it was felt necessary.

**General comment (contd.):**

Mat & Meth

In developing predictive models (simulators) to be applied in health, one needs to consider, first of all (among other variables) the benefits for the patient(s) which needs to be measured based on ‘physiological’ parameters. The M&M described in detail, which variables (n= 7, in this case) are of interest, and how they’ve been calculated (which is of the utmost relevance) for both the mechanical and the biological components. Notably, their model showed a correlation close to 1 (R=0.99, SE=0.02).

One of the most relevant aspects is that the authors considered, in their biological component, variables such as angiogenesis, ossification, chondrogenesis (formation of cartilage), calcification, etc., which are fundamental, as these occur in the ‘real’ physiological environment.

——

In their results, the authors describe the callus healing of both types of plates-techniques analysed (LP vs DCL) with their simulation model. Having stated previously, that the latter is the standard. From the clinical perspective, the variables compared between both models, are relevant, such as bone and cartilage concentration, blood perfusion, etc. Outstanding, is the fact that they present this comparison over space (1) and time (2). First, in a real scenario, fractures are not bi-dimensional, therefore a 3D model (as presented here) is more adequate. Second, a static screenshot of the changes might be also hard to analyse PROGRESSION/EVOLUTION of the healing process. Again, in a real patient, clinicians we need to evaluate how this improves in time. For this reason I found the model and the variables compared, very convenient.

Although described in human patients, this model can also be explored/applied in veterinary fractures which are physiologically and in various aspects, similar to those occurring in human patients. This might also be mentioned in their conclusions, should the authors agree on this. Moreover, to move on clinical trials (human), most studies are at first, tested on animal models (also known as clinical trial Phase 0)

**[Response]:** The authors gratefully acknowledge the reviewer’s thoughtful feedback on the rationale and outcome of the study. That some of our predictions accord well with the clinical observations is both encouraging and satisfying at the same time. The authors would also like to thank the reviewer for such detailed clinical insights which will help us immensely while carrying out an animal study in future for a full-fledged validation of the entire scheme. The authors also agree completely to the suggestion that this model can also be explored/applied in veterinary fractures which are physiologically and in various aspects, similar to those occurring in human patients. Realising the importance of this suggestion, the following sentence has now been added as a concluding remark in the revised manuscript (Page 14, Lines 23-25):

***“The model can also be explored/applied in veterinary fractures which are, physiologically and in various aspects, similar to those occurring in human patients.”***

**General comment (contd.):**

Results:

Post operative analysis of the three main components in the ROI, (1) blood perfusion, (2) cartilage concentration and (3) bone concentration, revelaed minor differences along the healing period (8 weeks), no apparent difference between LP and DCP was observed by the end of week 8. The authors furhter support this by breaking down the distribution of the different types of tissue along the healing period with no difference between LC and DCP in the percentage of woven, cartilage and connective tissue. Moreover, theses results were further comapred with two other studies, one is a model and the other an animal experiment. Here the authors noted accurately, that the present study showed an IDENTICAL trend to that study of Lacroix, et al. 2002. In medicine and clinical trials, trends are more important than isolated values, since htey predict or help to estimate a prognosis or the clinical outcome (which we always have to tell to our patients or their relative).

Differences with the animal model were more apparent, however, the authors highlighted the role of the input parameters, in the bone healing progression. As I mentioned above, one of the main powers of these studies, is that irrespective of their caveats given that they are models, is that one the one hand are cheaper and quicker to perform, and more importantly, that they provide information towards the different components that might be involved in a 'real bilogical/medical/clinical' scenario, in which case, there is always other factors that cannot be measured. Importantly, the authors contempalted the possibility of a in vivo study in the future. With regard to the predictive value of this model and the 'trend' discused above, this point was raised (and corrected) by previous reviewers (Page no. 9, line: 22-25), here they clarify that their model is in good agreement with animal experiments.

**[Response]:** The authors thankfully acknowledge the comments and the commendations. This will encourage us to take up more advanced studies in near future.

**General comment (contd.):**

I think that the second title (after concerns were raised by one reviewer) fits better with the study, HOWEVER, I would condition the approval of the manuscript for publication in PLos ONE as long as the word 'MODEL' is included in the NEW title. This is of the utmost relevance, precisely to avoid overinterpretation of their results. And to let the reader know, from the very begining that this study is based in a model (mostly) and not experimental data. This is important, since as it can be noted across the entire manuscript, the workd model is used abundantly.

**[Response]:** The authors acknowledge the above comment, and fully subscribe to the view that the word ‘MODEL’ should be mentioned in the title for better readability and understanding. Based on the suggestion, the title has now been revised as follows:

***New Title: A preclinical model of post-surgery secondary bone healing for subtrochanteric femoral fracture based on fuzzy interpretations***

***Old Title: Phenomenological prediction of post-surgery secondary bone healing for subtrochanteric femoral fracture based on fuzzy interpretations***

**General comment (contd.):**

I also ackowledge the value of the corrections and additional information that the authors amended after suggestions made by reviewers (or explain furhter when this was not possibly, as in the case of providing references given that no previous studies have been addressed the influence of screw type in fracture healing).

Some points were raised related to the use of passive/active voice, I agree with the aithors that in many aspects in scientific writing, we are encouraged to use passive voice. Other concerns of the meaning of language and jargon were raised too, but to me, the vocabulary was clear or when I had to, I searched for the meaning in either graphs or elsewhere and I could find it (at least in the revised form that I revised). Likewise, I noted that authors ammended and provided all the concerns from previous reviewers (data, clarity, references, etc), agreeing in most cases, which reflects their flexibility and scientific openess to discuss their work, which is also of great value. However, I had to agree with both previous reviewers that in some aspects the manuscript was not clear (maybe even less easy in its initial form) and that information of value was missed. Possibly some of this issues are down to the very technical terminology.

**[Response]:** The authors are grateful for the compliments from the esteemed reviewer. As for clarity in certain parts of the manuscript, the authors have now addressed most of those concerns by revising (i.e., changing from passive sense to active sense) the Results section as per Reviewer-1’s suggestions.
